# A Case of Diffuse Alveolar Hemorrhage Associated with High-Titer of MPO-ANCA Demonstrating Cytoplasmic Staining Pattern

**DOI:** 10.1155/2019/6074792

**Published:** 2019-12-12

**Authors:** Jihad Ben Gabr, Hiroshi Kato

**Affiliations:** Division of Rheumatology, SUNY Upstate Medical University, 750 East Adams Street, Syracuse, NY 13210, USA

## Abstract

Diffuse alveolar hemorrhage (DAH) is a life-threatening complication of ANCA-associated vasculitis (AAV) that requires urgent recognition and treatment. A presumptive diagnosis is often rendered without histopathology if concordant positivity of ANCA by indirect immunofluorescence (IIF) and ELISA assays, i.e., P-ANCA+/myeloperoxidase (MPO) Ab+ or C-ANCA+/proteinase-3 (PR3) Ab+, is documented in the context of pulmonary-renal syndrome or rapidly progressive glomerulonephritis. In this respect, the discordance between IIF and ELISA assays poses a diagnostic challenge in the absence of convincing histopathology and involves the risks of delaying the implementation of timely immunosuppressive therapy. Here, we report a 74-year-old woman who developed DAH and was found to have a high titer of MPO-ANCA exhibiting cytoplasmic staining on IIF, i.e., MPO-C-ANCA. The literature suggests that the availability of distinct epitopes on the MPO molecule dictates the perinuclear versus cytoplasmic staining pattern, which potentially explains the discordance between ELISA and IIF assays. Her DAH was controlled in association with seven sessions of plasmapheresis, methylprednisolone 1 gram daily for 3 days followed by 1 mg/kg/day, and rituximab. This case exemplifies the importance of consideration of pretest probability of suspected diagnosis that would realize a plausible interpretation of seemingly inconsistent serological profile and its effective incorporation into the diagnostic reasoning.

## 1. Introduction

DAH or pulmonary-renal syndrome is one of the rheumatological emergencies that requires urgent recognition and treatment to prevent fatality. The diagnosis of AAV is often rendered without histopathology when MPO- or PR-3-ANCA is documented in the context of pulmonary-renal syndrome or rapidly progressive glomerulonephritis [1]. MPO-ANCA usually exhibits a perinuclear staining pattern on IIF which is attributed to redistribution of positively charged antigens to the negatively charged nucleus as a result of ethanol fixation [2]. On the contrary, PR-3-ANCA gives rise to a cytoplasmic staining pattern. In this regard, the discordance between IIF and ELISA assays was documented in 1% cases of AAV in the EUVAS study [3]. Here, we present a case of DAH associated with a high titer of MPO-ANCA that demonstrated cytoplasmic staining on IIF. Following the case presentation, we will review the available literature that provides a potential explanation for such a discordant immunological profile.

## 2. Case Presentation

A 74-year-old Caucasian woman was admitted to our hospital 4 months prior to the current presentation for abrupt onset of purpura involving all limbs. The patient carried a diagnosis of polymyalgia rheumatica for which she had taken at least 2 mg of prednisone per day over a decade. Workup showed a positive ANCA demonstrating a cytoplasmic staining pattern at 1 : 1280 dilution and MPO-ANCA at 261 AU/ml (reference range: 0–19 AU/ml). The rash resolved in association with 60 mg of prednisone, which was tapered to 2 mg over two weeks. Three weeks later, the patient was admitted to the hospital for respiratory failure and was found to have multifocal pulmonary opacities. A presumptive diagnosis of multifocal pneumonia eliciting COPD exacerbation was made, and the patient was treated with antibiotics and intravenous methylprednisolone 40 mg twice daily, which led to remarkable improvement of pulmonary status. During the hospital stay, she received heparin injection for deep venous thrombosis prophylaxis and subsequently developed thrombocytopenia concurrently with bilateral pulmonary emboli. Such a clinical course and positive HIT antibody established the diagnosis of heparin-induced thrombocytopenia, and the patient was started on fondaparinux. Rheumatology consultation was not sought during the foregoing admissions.

A week prior to the current presentation, the patient had developed worsening dyspnea associated with nonproductive cough. In addition, the patient had developed polyarthralgia affecting the small joints in the hands and knees that aggravated in the morning and was associated with at least one hour of morning stiffness. She had had persistent microscopic hematuria at least over the prior 4 months, but her renal function had remained preserved throughout the clinical course. She had not had hearing loss, vertigo, alopecia, change of vision, ocular pain or hyperemia, epistaxis, recurrent sinusitis, mucosal ulcers, photosensitive rash, pleurisy, hemoptysis, postprandial abdominal pain, Raynaud's phenomenon, cutaneous ulcers, muscle weakness, or paresthesia. There was no history of seizures, stroke, coronary artery disease, or miscarriages. Her past medical history included hypertension, type 2 diabetes mellitus, osteopenia, chronic obstructive pulmonary disease (COPD), and stage II nonsmall cell lung cancer status after left lower lobectomy two years prior to the current presentation, for which she was receiving 2 liters of oxygen via a nasal cannula. Her relevant medications upon admission included allopurinol, minocycline, prednisone 2 mg per day, and fondaparinux. The indications for allopurinol and minocycline were unclear despite our thorough history taking and communication with her primary rheumatologist. The patient had allergies against clarithromycin and cephalexin. There was no family history of autoimmune diseases.

On examination, the patient was in moderate respiratory distress. The temperature was 37 degrees, the blood pressure was 130/93 mmHg, the pulse was 120 beats per minute, and the oxygen saturation was 98%, while she was using 4 liters of oxygen via a nasal cannula. Conjunctivae were pale, but not icteric. Her nasal and oral mucosae were normal. There was no tenderness over the sinuses. Superficial lymph nodes were not palpable. There were diffuse rhonchi in both lung fields. The patient was tachycardic, but was in regular rhythm. There was no pericardial friction rub, murmur, or gallop. There was no abdominal tenderness or organomegaly. There was no rash. There was tenderness over the bilateral proximal phalangeal, metacarpophalangeal, and radiocarpal joints. Her lower extremities showed mild pitting edema. The remainder of the physical and neurological examinations was unremarkable.

Laboratory studies showed a leukocyte count of 9800/*μ*l (reference range: 4100–11,000/*μ*l; reference range is provided in the parentheses in the following laboratory studies), eosinophil count 100 ul (0–500/ul, 1.3% of total leukocytes), hemoglobin 8.9 g/dL (12–16 g/dl), and platelet count 189,000/*μ*l (150,000–450,000/*μ*l). Her erythrocyte sedimentation rate was 110 mm/h (0–30 mm/h). PT-INR was 1.59. Chemistries showed BUN of 16 mg/dL (7–24 mg/dL), creatinine 0.99 mg/dL (0.6–1.0 mg/dL), aspartate aminotransferase 12 U/L (11–39 U/L), alanine aminotransferase 19 U/L (12–78 U/L), alkaline phosphatase 86 U/L (45–117 U/L), albumin 2.4 g/dL (3.2–4.5 g/dL), total bilirubin 1.6 mg/dL (0–1.0 mg/dL), lactate dehydrogenase 570 U/L (84–246 U/L), and haptoglobin 160 mg/dL (30–200 mg/dL). Arterial blood gas showed pH 7.45, PaO_2_ 58 mmHg, PaCO_2_ 32 mmHg, HCO3 21.4 mmol/L, and SaO_2_ 88.5% on 50% FiO_2_. Anti-nuclear antibody (ANA) indirect immunofluorescence assay was negative. ANCA was positive at 1 : 1280 dilution and exhibited cytoplasmic staining pattern. MPO-ANCA was strongly positive at 114 AU/ml (0–19 AU/ml), whereas neither proteinase 3 nor the glomerular base membrane antibody was detected. C3 and C4 were 145 mg/dL (90–180 mg/dL) and 34 mg/dL (10–40 mg/dL). Neither the rheumatoid factor nor cryoglobulin was identified. Anticardiolipin and beta-2 glycoprotein antibodies were negative. Hepatitis B and C serologies did not suggest past exposure or immunization. Serum protein and immunofixation electrophoreses did not show paraproteins. Urine microscopic exam showed 10–25 white blood cells per high-power field (hpf) and 25–50 red blood cells per hpf. There were no dysmorphic erythrocytes or erythrocyte casts. A spot urine protein to creatinine ratio was 0.55. A CT of the thorax showed centrally located multifocal opacities.

Upon admission, the patient was started on imipenem and vancomycin. Given the progressively worsening hypoxemic respiratory failure, the patient was transferred to a medical ICU on the 3^rd^ hospital day, when her trachea was intubated and mechanical ventilation was started. Following the intubation, bloody secretion was noted in the endotracheal tube and her hemoglobin dropped to 5.9 g/dL on the 5^th^ hospital day. Fondaparinux was discontinued. Such a clinical course, strongly positive ANCA, and the foregoing findings on CT raised a concern for diffuse alveolar hemorrhage as a manifestation of pulmonary vasculitis, and the patient received methylprednisolone 1 gram daily for 3 days followed by 40 mg twice daily, which was equivalent to prednisone at 1 mg/kg/day. Her allopurinol and minocycline were discontinued. The patient underwent bronchoalveolar lavage (BAL) on the 6^th^ hospital day, which confirmed the diagnosis of DAH, but did not yield any infectious organisms. A renal biopsy was performed on the 9^th^ hospital day. The specimen contained up to 40 glomeruli, of which 14 were sclerosed. The sclerotic glomeruli were clustered in a region of scar with surrounding atrophic tubules, interstitial fibrosis, and chronic inflammation. The remaining glomeruli showed no increase in the mesangial matrix or mesangial cellularity. The glomerular capillaries were patent without endocapillary hypercellularity. The glomerular capillary walls were normal on periodic acid-Schiff and silver stains. There was only mild interstitial fibrosis and tubular atrophy involving <25% of the cortical area outside the areas of the scar by the Masson trichrome stain. Arterioles showed mural thickening and small foci of hyalinosis. At least 7 arteries were represented and several showed moderate intimal sclerosis. Immunofluorescence microscopy showed trace mesangial staining for IgG, IgM, IgA, C3, C1q, kappa, and lambda, all of which was considered nonspecific. Electron microscopy showed no immune-type dense deposits. The mesangium showed mild matrix increase. The glomerular basement membranes demonstrated normal thickness. The podocyte foot processes were intact. Collectively, there was evidence of moderate nephron loss and parenchymal renal scarring associated with hypertensive-type vascular sclerosis, whereas there was no evidence of ANCA-associated inflammatory process.

The diagnosis of microscopic polyangiitis (MPA) was established based on the clinical features including polyarthralgia associated with inflammatory features, purpura, and DAH, as well as strongly positive MPO-ANCA. In association with the steroid pulse therapy, her DAH was better controlled as evidenced by improving oxygenation and stabilization of hemoglobin and hematocrit; however, the patient remained ventilator dependent. As such, she was started on plasmapheresis on the 14^th^ hospital day and received a total of 7 sessions of treatment in the ensuing two weeks. Immediately after the 2^nd^ and 72 hours prior to the 3^rd^ session of plasmapheresis, the patient received the 1^st^ dose of rituximab at 375 mg/body surface area m^2^. Following the two sessions of plasmapheresis, her pulmonary status markedly improved and she was extubated on the 17^th^ hospital day. Following completion of 7 sessions of plasmapheresis, she received additional 3 doses of rituximab at 375 mg/body surface area m^2^. On the 35^th^ hospital day, the patient was discharged to a rehabilitation facility on 2 liters of oxygen via a nasal cannula. The patient's arthralgia resolved in association with the foregoing immunosuppressive therapy. In the ensuing 9 months, her MPA remained in remission, which allowed her prednisone to be tapered to 5 mg per day.

## 3. Discussion

The EUVAS study showed that immunoassays performed better than IIF assays [3] and prove to be more specific than those reported by Hagen et al. [4]. Thus, this study established the basis of the 2017 International Consensus [5] which proposed high-quality immunoassays as the primary screening method for suspected cases of AAV without the need for IIF. However, these results must be interpreted cautiously. The EUVAS study excluded patients with inflammatory bowel disease and autoimmune liver disease from the control group, whereas Hagen et al. included patients with inflammatory bowel disease. Given the high prevalence of p-ANCA in the foregoing disease entities [6–8], such a difference in the control populations likely contributed to the higher specificity in the EUVAS study.

MPO-ANCA typically exhibits a perinuclear staining pattern, i.e., p-ANCA pattern, which is considered an artifact attributed to the redistribution of positively charged antigens to the negatively charged nucleus as a result of ethanol fixation [2]. We clarified with the laboratory that the leukocytes had been fixed in ethanol but not formalin in our case. Thus, the implication of MPO-ANCA showing cytoplasmic staining pattern, MPO-C-ANCA, was unclear. Such discordance appears to be uncommon as only 2 cases of MPO-ANCA exhibiting C-ANCA pattern and 1 case of PR-3-ANCA demonstrating P-ANCA pattern were documented out of 251 cases of AAV in the EUVAS study [3]. In this regard, the available literature suggests the presence of two distinct epitopes on the same MPO molecule that give rise to perinuclear and cytoplasmic staining patterns [9]. In this study, two different monoclonal antibodies that recognize distinct MPO epitopes, 2B11 and MPO-7, were utilized to determine the specificity of MPO-ANCA exhibiting perinuclear (MPO-P-ANCA) and cytoplasmic (MPO-C-ANCA) staining patterns. Sera from 2 out of 3 patients with MPO-C-ANCA nearly completely inhibited the binding of 2B11 to MPO, whereas sera from 7 out of 11 patients with MPO-P-ANCA substantially blocked the binding of MPO-7. Likewise, samples from 2 out of 3 patients with MPO-C-ANCA bound to a 40 kDa component of MPO on immunoblotting, whereas only those from 2 out of 11 patients with MPO-P-ANCA showed such reactivity. Further, MPO-C-ANCA and MPO-P-ANCA epitopes were shown to be present in all three major isoforms of MPO by ion-exchange chromatography [9]. These findings suggest that the availability of individual epitopes dictates a distinct immunofluorescence pattern.

From a diagnostic reasoning point of view, this case exemplifies the importance of pursuing a unifying diagnosis upon encountering a patient who has developed symptoms in multiple organ systems over a short time period. Our patient developed purpura, polyarthralgia associated with inflammatory features, and recurrent pneumonitis over a course of 4 months, all of which were amenable to steroid treatment; however, it was not until she developed DAH when the diagnosis of ANCA-associated vasculitis was finally entertained. As an example, noninfectious etiologies were not considered as the cause of multifocal pulmonary opacities 3.5 months prior to the current presentation. However, if all of the clinical features were considered in the context of the high titer of MPO-ANCA, an earlier diagnosis would have been rendered.

As noted earlier, it was unclear why the patient had been on allopurinol and minocycline. Our evaluation did not disclose any historical elements or clinical findings suggestive of gout. Regardless, as both minocycline and allopurinol have been implicated as a trigger of AAV [10, 11], it was prudent to discontinue these agents without clear indications. However, we reason that minocycline was likely not the precipitating factor of her vasculitis. With regard to the clinical manifestations of vasculitis, minocycline is most commonly associated with arthritis, livedo reticularis, and polyarteritis nodosa- (PAN-) like medium vessel vasculitis [12–19]. As is the case in PAN *per se*, pulmonary involvement appears to be uncommon in minocycline-induced vasculitis [14, 16, 17, 20]. A case of minocycline-induced pauci-immune crescentic glomerulonephritis was reported; however, the argument for causality was dampened by that the patient had been treated with a pulse dose of steroid, plasmapheresis, and cyclophosphamide [21]. In most but not all cases of minocycline-induced vasculitis, the clinical manifestations resolved upon drug discontinuation [12, 14, 22–24], whereas our patient's pulmonary vasculitis did not follow such a trajectory and necessitated aggressive immunosuppressive therapy. Two cases of allopurinol-induced AAV have been described [11, 25]. Both cases were characterized by markedly elevated MPO-ANCA [11, 25]. The first reported case presented with leukocytoclastic vasculitis which resolved along with normalization of ANCA in association with drug discontinuation [25]. The second case was characterized by pulmonary hemorrhage and kidney involvement requiring intense immunosuppression although lung involvement was found to be significantly more common in AAV not associated with relevant drug exposure in this case series [11]. Although allopurinol might have played a role in our case, the causality cannot be confirmed given the paucity of published data and the concurrent immunosuppressive therapy the patient received in addition to the drug discontinuation. Finally, we would like to point out that there is no literature implicating these drugs as an etiology of MPO-C-ANCA or discordance between IIF and ELISA ANCA assays although positive P-ANCA or MPO-ANCA was reported in a large proportion of patients with minocycline-induced vasculitis or autoimmunity [10, 12, 18, 22, 23, 26] and all reported cases of allopurinol-induced AAV [11].

In terms of therapeutic management, the PEXIVAS study presented in the 2018 American College of Rheumatology meeting questioned the roles for plasmapheresis in AAV [27]. However, published studies supported the roles of plasmapheresis in those with severe renal disease [28, 29] or DAH [30]. Additionally, it appears to serve as an excellent rescue therapy in AAV [31]. Therefore, we reason that plasmapheresis remains integral to the management in selected cases of ANCA-associated vasculitis, and a decision regarding its use should be tailored to the individual clinical circumstances. While our patient's pulmonary disease exhibited steroid responsiveness, she remained ventilator dependent after steroid pulse therapy. In contrast, her pulmonary status markedly improved following two sessions of plasmapheresis. Therefore, plasmapheresis was clearly beneficial in our case.

In summary, we reported a case of pulmonary vasculitis associated with a high titer of MPO-ANCA that exhibited cytoplasmic staining on IIF. Our case reinforces a clinical pearl that it is imperative to take into account the pretest probability and gravity of suspected clinical conditions so that seemingly inconsistent laboratory findings would be incorporated into the diagnostic reasoning instead of being discarded. In this respect, a thorough scholarly review was profoundly effective in this case and allowed for a plausible interpretation of such inconsistency.
